# Large language models for autism: evaluating theory of mind tasks in a gamified environment

**DOI:** 10.1038/s41598-025-18608-4

**Published:** 2025-10-06

**Authors:** Christian Poglitsch, Anna Reiss, Selina C. Wriessnegger, Johanna Pirker

**Affiliations:** 1 Institute of Human-Centred Computing, Graz University of Technology, Graz, 8010 Austria; 2 Institute of Neural Engineering, Graz University of Technology, Graz, 8010 Austria; 3 Technical University of Munich, Munich, 80333 Germany

**Keywords:** Large Language Models, Gamification, Social interactions, Autism, Theory of Mind, Health care, Public health

## Abstract

Autism Spectrum Disorder often significantly affects reciprocal social communication, leading to difficulties in interpreting social cues, recognizing emotions, and maintaining verbal interactions. These challenges can make everyday conversations especially demanding. To support autistic people in developing their social competence and communication abilities, we propose an interactive game specifically designed to enhance social understanding. By incorporating gamification elements and a user-centered design approach, the application aims to balance clinical relevance with high usability, ensuring it remains accessible, engaging, and beneficial for anyone seeking to improve their social skills. Large Language Models have recently been assessed for their ability to detect sarcasm and irony within Theory of Mind tasks, showing performance comparable to that of trained psychologists. However, a significant limitation remains: their dependence on traditional “black box” AI architectures, which often lack explainability, interpretability, and transparency. This limitation is particularly concerning when people with and without Autism Spectrum Disorder use these models to learn and practice social skills in safe, virtual environments. This study investigates and compares the performance of Large Language Models and human experts in evaluating Theory of Mind tasks, providing a detailed comparative analysis. A total of 21 participants engaged with our game, and their responses were assessed by four human experts alongside GPT-4o. The results indicate that GPT-4o matches human experts in both adherence to instructional criteria and evaluation accuracy, with no statistically significant differences observed. These findings underscore the potential of LLMs to support scalable, always-available social training systems that are accessible from anywhere.

## Introduction

Autism Spectrum Disorder (ASD) can profoundly affect cognitive and social functioning, often leading to substantial and lasting challenges^1^. It is a lifelong condition that is frequently associated with a high prevalence of co-occurring psychiatric disorders, including depression, anxiety, attention deficit/hyperactivity disorder (ADHD), and emotional instability syndromes^2^. According to the American Psychiatric Association (APA)^3^, common autistic characteristics include: (1) impaired social perception (perspective taking), (2) impaired interpersonal social competence(an autistic person who speaks few or no words, gaze behavior, gesture, facial expression), and (3) compulsive, stereotyped thinking and behavior, along with narrowly focused interests. In ICD-10, autism spectrum disorders (ASD) are classified as ’pervasive developmental disorders’ (F84). Specifically, ICD-10 distinguishes between early childhood autism (F84.0), atypical autism (F84.1), and Asperger’s syndrome (F84.5).

**Fig. 1:**
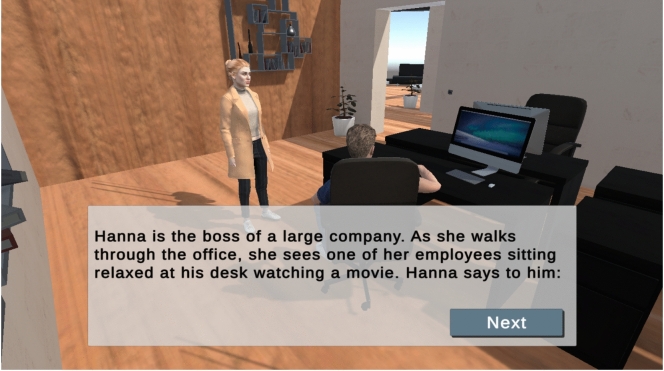
A game scenario where the user is tasked with detecting irony and sarcasm in a dialogue between Hanna and her employee.

With appropriate support and interventions, such as training social skills in virtual reality^4^ and the use of visual aids or assistive technologies,autistic people can improve their conversational skills^5,6^ and navigate social interactions more effectively. In this paper, our goal is to design virtual and safe scenarios to support autistic people in developing their social skills within a gamified environment, as a screenshot of the game illustrates in Fig. 1. Play is a fundamental context in which people develop social understanding and practice perspective-taking; however, autistic people often experience differences in symbolic and social play, which can affect the development of communication and Theory of Mind (ToM)^7^. Gamification, defined as the incorporation of game design elements into non-game contexts^8^, positively influences learning motivation^9^. To provide feedback, we plan to use Large Language Models (LLMs), which offer continuous access to extensive knowledge and can generate responses that simulate human-like interactions across a wide range of topics. LLMs are capable of explaining psychological methods, simplifying complex texts in plain language, and supporting self-explanation. We compare the ability of GPT-4o to evaluate responses in theToM tasks with the assessments provided by human experts specializing in clinical psychology. This study is based on the following research questions that examine how effectively LLM can support social learning forautistic people in a gamified environment.

- RQ1: How accurate can LLMs follow instructions to evaluate human feedback in gamified social training tasks?
- RQ2: How well can LLMs assess participants’ performance on Theory-of-Mind tasks relative to human expert evaluations?

## Related work

The following section reviews previous research on gamification in autism interventions, the integration of LLMs into gamified and healthcare contexts, and the relationship between ToM, gamification, and LLMs.

### Gamification and autism

Studies show that gamification is becoming increasingly popular in health education^10,11^. In healthcare, gamification improves user engagement by making health-related activities more interactive, motivating, and enjoyable. When games are enjoyable and aligned with evidence-based therapies, they can help bridge the gap between the recommended and actual amounts of therapy received byautistic people^12^. Engagement is further enhanced when gamification elements such as progression, feedback, and rewards are used to promote self-care behaviors, including knowledge acquisition, self-advocacy, and healthy lifestyle choices^13^.

### Large language models in gamification and autism

In game scenarios, LLMs demonstrate the potential to simulate human life by mimicking interactions among individuals in a virtual village^14^. Artificial Intelligence (AI) agents, such as chatbots and virtual assistants, can be used in healthcare to provide support and distribute information using healthcare training data^15^. An accurate representation of virtual humans is crucial for effective training in social skills within a virtual environment. This allows users to gain a deeper understanding of empathy, especially when the virtual character also provides empathic feedback^16^. The use of virtual avatars in assistive learning has also gained considerable attention in recent research. Systematic reviews have shown significant improvements in social skills, emotional abilities, daily living skills, communication, and attention through interactions with such avatars^5,6^. A gamified online role-play demonstrated significant potential in enhancing self-perceived confidence and awareness in teledentistry, where a professional actress was trained to role-play a patient with a dental problem^17^. Experimental findings revealed that the integration of empathic modules, which simulate emotional states and provide clarification for each response, significantly improved the ratings of empathic listening and emotional intelligence scores, leading to a higher level of trust^18^.

The potential benefits of using Large Language Models (LLMs) include question answering, decision support, information retrieval, and multimodal medical applications^19^. LLMs exhibit strong potential in healthcare applications due to their ability to follow instructions, perform advanced natural language processing, and adapt to contextual learning. Their integration into clinical workflows can support decision-making processes, improve patient care, and help reduce medical errors^15^. LLMs have demonstrated the ability to simulate mental state reasoning, mirroring human performance inToM assessments^20–23^.

### Theory of mind and its relationship to gamification and large language models

They also show sophisticated decision-making and reasoning skills, effectively solving the challenges traditionally used to evaluate ToM in humans. These capabilities are the result of interdisciplinary advances in psychology, communication, and artificial intelligence research^24^. ToM refers to the cognitive capacity to recognize that others have thoughts, feelings, beliefs, desires, and intentions that differ from their own. Research has shown that people on the autism spectrum often experience delays or impairments in the development of ToM abilities. To systematically assess ToM skills, researchers have developed a range of specialized tasks, including the *Faux Pas Test*, *Irony Tasks*, *Strange Stories*, *False Belief Tasks*, and the *Hinting Task*^22,25^. These tasks are typically composed of short narrative vignettes that describe social interactions, followed by one or more questions that require participants to interpret or predict the behavior of a character. Correct responses require the ability to adopt another person’s perspective and infer their mental state. These difficulties can lead to significant challenges in daily life, including increased stress and limitations in social interactions. Unlike neurotypical people, whose ToM skills are typically intuitive and automatically applied,autistic people often rely on more conscious and deliberate reasoning to interpret others’ mental states. Studies suggest that ToM abilities can be improved through training interventions^22^. LLMs have demonstrated the ability to simulate mental state reasoning, mirroring human performance in social tasks^20,21^. These capabilities are the result of interdisciplinary advances in psychology, communication, and artificial intelligence research^24^. In addition, LLM have shown to provide mostly accurate responses to a variety of medical questions, as evaluated by academic physician specialists^26^, suggesting greater utility in clinical support roles. Recent studies have shown that LLMs can perform on par with human participants, or even outperform, in specific ToM tasks^20,27^. In a study where a clinical psychologist assessed model-generated responses without knowing their source, GPT-4’s outputs were rated as more clinically relevant and empathetic than those produced by ChatGPT.

LLMs have limitations, including the possibility of inaccuracies or a ’hallucinated’ output resulting in factual incorrectness and misinformation^28^. Therefore, LLM requires substantial human supervision, professional leadership, and judgment^29^. There are also requirements for continued research and development to optimize large language models for clinical use in mental health care settings because the results demonstrated a significant difference in performance between models^30^. Their vulnerability to small perturbations in the provided prompts raises concerns about the robustness and interpretability of their observed successes^20^. These findings highlight the need for ongoing research and refinement to optimize large language models (LLMs) for application in mental health care settings^30^.

## Method

This study aims to develop a series of interactive social scenarios designed to Helpautistic people improve their social and conversational skills in a gamified, virtual, and safe environment and to evaluate whether LLMs can reliably assess responses to ToM tasks. If successful, this could pave the way for automated feedback systems, particularly to support people in training their social abilities. To assess the performance and potential of LLMs in providing feedback on ToM tasks, expert evaluations will be compared to those generated by LLMs. The degree of agreement and divergence between the human and AI assessments will be quantitatively analyzed.Inclusion criteria for experts required professional training in psychology, therapy, or medicine, with specialized knowledge of autism and practical experience in administering or evaluating ToM tasks. For participants, inclusion criteria required being at least 16 years of age, providing informed consent; both autistic and non-autistic people were included, with recruitment conducted through local autism centers, support groups, and the university campus.

The study was carried out in full compliance with ethical guidelines and standards. All procedures were reviewed and approved by the Ethics Committee of the Graz University of Technology (approval number EK-51/2024) on January 17, 2025.

### Experts

To complement the automated evaluation with expert judgment, we included human experts from a local autism center. All experts completed a short questionnaire on their background knowledge of ASD and their experience evaluating ToM tasksThe experts were asked about their profession, the length of time they have been working in the field, whether they work with autistic people, and whether they use ToM tasks in their work. This ensured that all participants had relevant expertise in both domains. We recruited experts in the field of autism, including therapists, physicians, and clinical psychologists, all of whom have specialized knowledge and extensive experience working with people on the autism spectrum. In total, we were able to recruit three clinical psychologists and one psychologist with professional experienceas shown in Table 1. Each expert evaluated 50% of the tasks of the participants. Their evaluations served as a reference point for validating the performance of the language models and for interpreting the qualitative aspects of the participant responses.

**Table 1:** Overview of experts’ professional background and experience.

Profession	Years of Experience	Work with ASD	Use ToM Tasks
Psychologist	25 years	Yes	Yes
Clinical Psychologist	5 years	Yes	Yes
Clinical Psychologist	4.5 years	Yes	Yes
Clinical Psychologist	15 years	Yes	Yes

### Participants

Recruitment was carried out through local autism centers, autism support groups, and the campus of Graz University of Technology. All participants provided their informed consent via an online survey and received compensation in the form of entry into a lottery for a voucher from a local chocolate factory. Until publishing a total of $N = 25$ participants participated in the study, of whom $N = 21$ completed all tasks. Four participants did not finish the game. The participants’ ages ranged from 16 to 58 years, with a mean age of 31.04 years. The sample included 10 women, 12 men and 3 people who identified themselves as of another gender. Seven participants reported having a clinical diagnosis of ASD.

### Study design

**Fig. 2:**
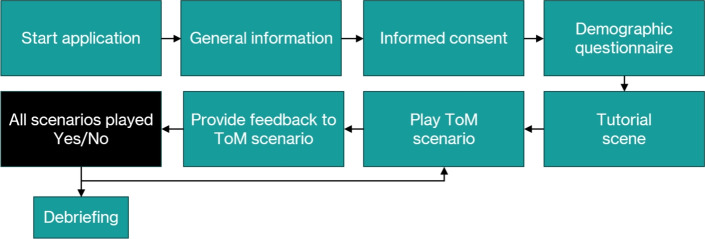
The diagram illustrates the study workflow from the player perspective.

Participants are provided with a link to complete the study remotely.The workflow from the participants’ perspective is shown in Fig. 2, with the duration for each step:

1. Participants access the online questionnaire through a link provided in the recruitment brochure or email.
2. Upon opening the questionnaire, participants are greeted with a brief introduction to the study, including an explanation of its purpose and an informed consent form. *(Estimated duration: 5 minutes)*
3. Participants complete a demographic questionnaire. *(Estimated duration: 5 minutes)*
4. This is followed by a series ofToM tasks. *(Estimated duration: 25–30 minutes)*
5. Finally, participants receive a short debriefing, are given contact information for further questions, and are thanked for their participation. *(Estimated duration: 5 minutes)*

All collected data is stored anonymously on a secure server to ensure user privacy and data protection. After completion of data collection, the participants’ responses to ToM tasks were evaluated.

### Game design

**Fig. 3:**
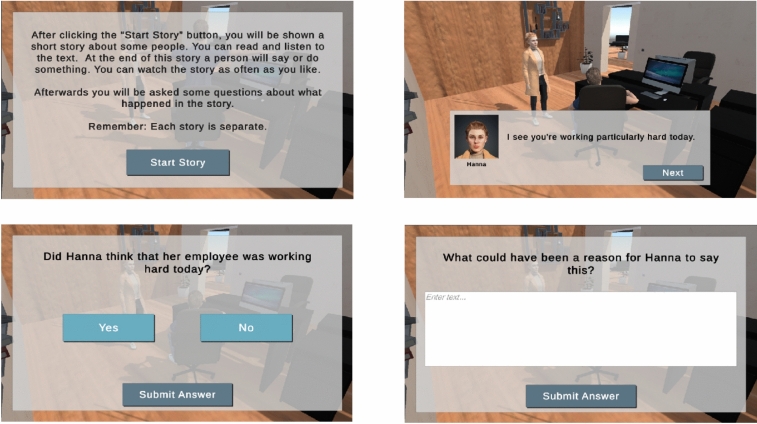
The top-left image displays the game instructions. The top-right image shows the task interface, where avatars narrate a story. The bottom-left image illustrates the graphical user interface for answering yes/no questions. The bottom-right image presents the interface for providing open-ended responses to the task-related questions.

Our framework, *Social Training* as shown in Fig. 3, utilizes the game engine Unity for visual rendering and user interaction, and integrates Reallusion avatars to model emotional expression. Feedback from participants was collected online using LimeSurvey. The game, compiled for WebGL, is accessible online via our server. The voice narration for the animated scenarios was generated using ElevenLabs.

The participants were presented with each story in the form of a short animated scene. They observed the characters interacting, listened to the narration (with distinct voices for each character and the narrator) and read the story as it appeared on the screen. The participants were allowed to replay the scenes as often as they wished. Once they felt prepared, they proceeded to answer the corresponding questions, presented sequentially. They could not revisit previous questions or replay scenes once they had started the questionnaire. The number and type of questions (yes/no or open-ended) varied by task category. However, each task ended with two comprehension questions designed to assess whether the participants had understood and remembered the factual details of the story; no interpretation was required for these items. Furthermore, each task included at least one question that addressed each of the following aspects: understanding nonliteral meaning or inference of hidden meaning, understanding social meaning, and prediction of the mental states of the characters involved. The responses of the participants to the tasks were evaluated by human experts and the GPT-4o language model. These evaluations were then compared to examine the potential differences between the human and AI assessments.

### Tasks

The study includes four types of theory-of-mind tasks, each targeting a specific aspect of social reasoning: 1) faux pas, 2) irony, 3) hint, and 4) strange stories (lie). Faux pas tasks involve socially inappropriate or awkward situations, irony tasks require the interpretation of non-literal language, hint tasks assess the ability to understand indirect suggestions, and lie tasks focus on detecting intentional deception. All stories and tasks were self-developed in collaboration with experts in clinical psychology to ensure their validity and relevance.

- **Social Faux Pas Task**
  - *Task 1 (with Faux Pas):* Camila buys cat food and mentions her new black cat to her colleague Kevin. Kevin makes an awkward comment about black cats being ugly and bad luck.
  - *Task 2 (without Faux Pas):* Caleb overhears two colleagues discussing cupcakes he brought on his first day and no inappropriate or awkward comment is made.
- **Irony Task**
  - *Task 1 (with irony):* Melissa sarcastically tells an employee watching a movie that he is “working particularly hard today.”
  - *Task 2 (without irony):* Kevin returns from shopping with lots of food. His wife comments positively, without irony, about having bought everything they need.
- **Hinting Task**
  - *Task 1 (with hint):* Camila mentions her puppy needing to go outside but hints for her husband to take the dog out because she is busy.
  - *Task 2 (without hint):* Caleb tells Melissa he has a headache and will take a painkiller which is a direct statement — no hinting.
- **Strange Stories Task**
  - *Task 1 (White Lie):* Kevin compliments new office chairs he actually dislikes, to avoid disagreeing with his boss Caleb.
  - *Task 2 (no White Lie):* Melissa tells her friend Camila that she loves lasagna, which is now true even though she disliked it as a child — no lie.

### Evaluation instructions

Each task has five to nine questions that are evaluated. One point was awarded for each correct answer and zero points for each incorrect answer. Partial points were not allowed. The scores are shown in Table 2.

**Table 2:** Maximum achievable points per task and total in the study.

Category	Task	Max. Points
Social Faux Pas	with Faux Pas	9
	without Faux Pas	9
Irony	with Irony	5
	without Irony	5
Hinting	with Hint	5
	without Hint	5
Strange Stories	White Lie	6
	No White Lie	6
**TOTAL per participant**		**50**

### Large language model prompt instruction

To design effective prompts for the assessment system, we adopted a structured approach comprising three key components: *1) Instruction*, *2) Story*, and *2) Evaluation*. For each task, GPT-4o was prompted to assess participant responses according to predefined scoring criteria. The following is the list of instructions given to the model:

1. **General Instruction:**
  A instruction text is included at the beginning of the prompt. This instruction outlines the structure of the study, the task categories, the type of questions, and the scoring guidelines.
2. **Task Story:**
  The complete text of the corresponding task story (including task type, narrative, and questions) is appended.
3. **Evaluation:**
  The participant’s responses for the current task are retrieved from the corresponding Excel sheet. Each response is extracted and formatted into question?: answer pairs using the delimiter “?:”. These pairs are presented as a structured list within the prompt.
  The evaluation instruction then defines the expected output format: a space-separated list of binary scores (0 or 1), corresponding to the participant’s responses to each question.

The instruction requires the LLM to output evaluation scores on the last line of its response, formatted as a space-separated sequence of binary values (e.g., 0 1 1 0). The number of values must match the number of questions. In the post-processing step, a script extracts the final line of the LLM output, converts it into a list of integers, and writes the scores into the corresponding columns of a CSV file.

## Results

This section presents the results of the comparative analysis of GPT-4o and human expert evaluations across allToM tasks. It highlights how well the model performs in terms of scoring accuracy and consistency.

### Large language model

For the evaluation, we used GPT-4o. The total computational cost for processing all participant responses was $0.24 USD. During the evaluation phase, the model handled approximately 105,019K input tokens and generated 2,056K output tokens. The total sequential processing time for evaluating all responses from the 21 participants was 230.6 seconds, demonstrating that LLM-based assessment can be conducted efficiently and at minimal computational cost.

### Evaluation

Results were evaluated and coded by four human experts. To assess the differences in evaluation scores between the language model and human responses, Mann–Whitney U tests were performed for the entire dataset and for each individual category. The test results are summarized in Table 3. GPT-4o generated correctly formatted output for all tasks, achieving a 100% success rate in adhering to the specified response format. The average computation time per task was 0.7 seconds.As a preprocessing step, the scores provided by the experts were averaged.

**Fig. 4:**
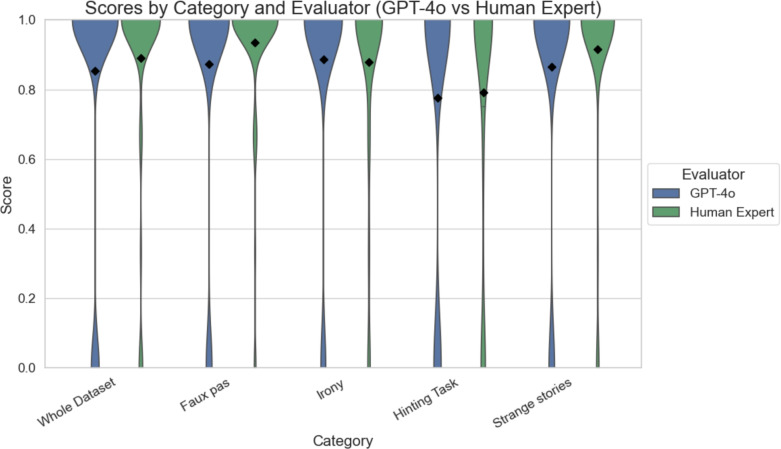
Comparison of evaluation scores across categories for GPT-4o and human experts. The violin plots illustrate the distribution of scores for each task type, where each response is scored as either correct (1) or incorrect (0), with black diamonds representing the mean values. Statistical analysis using the Mann–Whitney U test revealed no significant differences between GPT-4o and human experts across all categories—including the overall dataset, Faux Pas, Irony, Hinting, and Strange Stories—suggesting a high level of consistency between GPT-4o and trained human evaluators.

As shown in Fig. 4, scores across different categories are compared between GPT-4o and human experts. The violin plots illustrate the distribution of scores for each task type, with black diamonds indicating the mean values. Statistical differences were assessed using the Mann–Whitney U test.

**Table 3:** Mann–Whitney U test statistics comparing GPT-4o and human expert evaluation scores across allToM task categories. No statistically significant differences were observed, indicating strong alignment between model and expert assessments.

Category	U Statistic	p-value
Whole Dataset	548509.5	0.7495
Faux pas	69918.0	0.3711
Irony	23163.0	0.1439
Hinting Task	22304.0	0.7834
Strange Stories	30482.0	0.1635

**Whole dataset**

A Mann–Whitney U test conducted on the full dataset revealed no statistically significant differences between GPT-4o and expert-generated evaluation scores ($U = 548509.5$, $p = 0.7495$). This indicates a high degree of consistency between the assessments produced by GPT-4o and human evaluators across all ToM tasks and participants. These results support the hypothesis that GPT-4o can reliably assess open-ended social cognition tasks, performing on par with trained clinical psychologists.

**Faux Pas Task**

In the faux pas category, the comparison yielded $U = 69918.0$, $p = 0.3711$, indicating no significant difference between GPT-4o and human ratings. This result indicates a high level of agreement when it comes to evaluating pragmatic reasoning and identifying socially inappropriate behavior.

**Irony Task**

For the irony task, results showed $U = 23163.0$, $p = 0.1439$. Although not statistically significant, the lower p-value may indicate greater variability in the interpretation of sarcasm and non-literal meaning. Nevertheless, GPT-4o generally aligned with expert evaluations.

**Hinting Task**

The hinting task, which tests the recognition of indirect suggestions, showed a high degree of agreement between evaluators ($U = 22304.0$, $p = 0.7834$), indicating that GPT-4o accurately interpreted subtle communicative cues similar to human experts.

**Strange Stories Task (White Lies)**

In the strange Stories task, focusing on mental state reasoning and prosocial deception, the comparison yielded $U = 30482.0$, $p = 0.1635$. No significant difference was observed, further reinforcing GPT-4o’s capacity to evaluate nuanced social reasoning on par with human judgment.

## Discussion

The results are promising, as GPT-4o was able to evaluate all tasks submitted by users and correctly interpret them within the appropriate context. Previous studies have shown that large language models (LLMs) perform well on ToM question batteries^20,31,32^. The experts and GPT-4o consistently evaluated the responses in a similar manner, demonstrating that the language model can effectively mimic expert behavior. It is also worth noting that the response scores were generally high, suggesting that the tasks may have been somewhat too easy, an aspect that should be considered in future work. Other studies^33^ suggest that the pretrained knowledge of LLMs is sufficient for evaluating tests or exams. However, fine-tuning is generally necessary and human oversight remains essential. Our results lead to a similar conclusion, showing that although LLMs solve exams, they are reasonably effective as graders^34^.

A notable advantage of LLMs over traditional rule-based game AI systems (e.g., finite state machines or behavior trees) is their capacity to understand context, nuance, and nonliteral language such as sarcasm or indirect communication. While conventional game logic excels at predictable and structured interactions, it lacks the flexibility to assess subtle mental state reasoning. LLMs, trained on extensive linguistic and conversational data, are better suited for dynamic and socially complex tasks such as interpreting irony or evaluating empathetic responses key elements inToM. Despite these advantages, critical challenges remain. First, LLMs are sensitive to prompt design. Slight variations in input formatting or instructions can lead to inconsistent output^33^. Although structured prompting was used to minimize this variability, it highlights the need for robust prompt engineering frameworks when deploying LLMs in sensitive applications such as psychological assessment or clinical support.

Our results highlight the potential of LLMs to support ToM tasks in a game-based environment. However, it is crucial to recognize that LLMs operate as “black box” systems, with internal decision-making processes that are not fully transparent. Before deploying such models in clinical or educational settings, rigorous evaluation is essential to ensure their reliability, fairness, and interpretability.

Ethical considerations are equally important. Data privacy and transparency are main concerns when using LLM in health-related applications. Although anonymization techniques can help mitigate data protection risks, the interpretability of AI decisions and the need for clear communication about system limitations remain essential to building trust, particularly when the target users are from vulnerable populations, such asautistic people.

The results presented above address our research questions:

- RQ1: How accurate can Large Language Models follow instructions to evaluate human feedback in gamified social training tasks?
  * The results show that all tasks produced correctly formatted answers when the input included a comprehensive prompt consisting of the instruction, the story, and the user’s response.*
- RQ2: How well can Large Language Models assess participants’ performance on Theory-of-Mind tasks relative to human expert evaluations?
  *The analysis showed that the GPT-4o evaluations were statistically indistinguishable from those of clinical psychologists in all categories of theToM task, including irony, hinting, white lies and social faux pas. The results suggest that LLMs are capable of reliably evaluating complex social cognition tasks in a structured, gamified environment.Our findings extend prior work on LLM performance in ToM tasks*^20,21^ *by demonstrating that these models not only achieve accuracy comparable to human participants but also align closely with expert evaluations when assessing real participant responses in a gamified environment. This is consistent with recent empirical studies showing that Large Language Models can approximate human-level grading across different domains*^35^. *From a practical standpoint, this indicates their potential as scalable tools for social training support, particularly where expert supervision is scarce.*

## Limitations

Although the results of this study are promising, several limitations must be acknowledged: 1) The sample size was relatively small (N = 21 participants completed), which limits the generalizability of the findings. The study is ongoing and future iterations with larger and more diverse populations—particularly those that allow comparisons between user responses—are expected to produce more insightful results.2) The evaluation relied on binary scoring, which is faster but does not capture nuanced responses. However, this simplicity makes it easier to incorporate game elements, such as high scores, in a gaming environment.3) Although GPT-4o demonstrated high agreement with expert evaluations, the LLM was not embedded into the game system in real time. All evaluations were conducted offline. This limits our ability to assess the responsiveness, latency, and user experience of an integrated system, which are critical factors for clinical and educational deployment.We decided to delay implementing real-time functionality until we have validated data demonstrating that the system works. This is particularly important when working with vulnerable user groups, such as autistic people. 4) LLMs are inherently opaque (“black box” systems), and their decisions are not always interpretable or robust. Minor changes in prompt language or task structure may influence their output. Although we used structured prompts and strict output formats to mitigate this, full transparency and explainability remain ongoing challenges.

## Conclusion

This study investigated the use of LLMs, specifically GPT-4o, to evaluateToM tasks in a gamified environment designed to support people training social situations. Our findings indicate that GPT-4o can provide evaluations that are statistically comparable to those of trained human experts in clinical psychology, with no significant differences observed in different tasks, including faux pas, irony, hinting, and white lies. The results suggest that LLMs, when properly guided by structured prompts and evaluation formats, are capable of reliably assessing complex social cognition tasks. This also supports the findings where LLMs were evaluated on their ability to answer ToM questions^20^. We extend these findings by evaluating the responses provided by real people. This paves the way for scalable, accessible, and continuous social skills training tools that can effectively complement existing therapeutic interventions. For a gamified environment, correct answers can be converted into high scores to enhance player motivation and engagement.

Although the current implementation does not yet feature real-time LLM integration, the technical evaluation of the GPT-4o output demonstrates its potential as a feedback engine in interactive learning environments. However, we emphasize the importance of continued validation, especially in clinical contexts, due to the inherent opacity of LLMs and their susceptibility to prompt sensitivity. This study contributes to the growing field of human-centered AI by demonstrating that LLMs can assist in socially meaningful tasks if properly constrained, validated, and used in collaboration with domain experts.

## Supplementary Information

Below is the link to the electronic supplementary material.

Supplementary Material 1

Supplementary Material 2

Supplementary Material 3

Supplementary Material 4

## Data Availability

The evaluation instructions, the expert questionnaire, the tutorial scenario, and all the story-based tasks used in this study are available at https://github.com/ChristianPoglitsch/AIAgents.

## Code availability

The code used for all analyses presented in the main manuscript, together with additional resources, is available at: https://github.com/ChristianPoglitsch/AIAgents.
