# Supplementary material for: Large language models for autism: evaluating theory of mind tasks in a gamified environment
Source: Sci Rep. 2025 Oct 6;15:34763. doi: 10.1038/s41598-025-18608-4 (PMC12501279; doi:10.1038/s41598-025-18608-4)
Supplement: Supplementary file 1 — Supplementary Material 1 [file 41598_2025_18608_MOESM1_ESM.pdf]

Scoring the faux pas task:

The Faux Pas test results should not really just be reported as an overall score. An overall score can be calculated, but there are several scores that should be reported to make clear where the respondent is having trouble Feel free to email me with questions (vestone@gmail.com), because it can seem complicated, but it's actually quite intuitive, and I and other researchers have found this is what works best.

Basically, use common sense. For each Faux Pas Story (stories 2, 4, 7, 11-16, and 18), the subject gets 1 point for each question answered correctly.

Faux Pas Detection

First question: "Did anyone say something they shouldn't have said?"

Faux pas stories:	Correct: Yes	Incorrect: No
Control stories:	Correct: No	Incorrect: Yes

Second question: "Who said something they shouldn't have said?"
Any answer that unambiguously identifies the correct person is correct, names are not necessary. E.g.,
story about calling little girl a boy: Mary (also acceptable: the neighbor)
story about crystal bowl: Anne (also acceptable: the hostess, or the woman who got married, etc.)
story about lawyers: Claire (also acceptable: the woman, or the woman in a bad mood, etc.)
story about curtains: Lisa (also acceptable: the friend)
story about cancer joke: Robert (also acceptable: the guy who came in late)
story about losing story contest: Jake (also acceptable: the guy who won)
story about spilled coffee: Tim (also acceptable: the guy who spilled his coffee)
story about new kid in school: Joe (also acceptable: Joe and Peter)
story about surprise party: Sarah (also acceptable: the woman who spilled the coffee)
story about pie: Joe (also acceptable: Kim's cousin)
Respondents who answer "no" to the first question don't get asked this question and score a 0 for this
one.

Scoring this 2nd question for the Control Stories is a bit more counterintuitive, because you give points for not answering, and take points off for answering. If they correctly say there was nothing inappropriate said, then they should not be answering this question. So, give them one point for each "who" question they do not answer on the Control Stories. If they answer the question, the answer will be wrong, so give them zero points for each "who" question they answer.

Understanding Inappropriateness

Third question: "Why shouldn't they have said it?"
For the Faux Pas Stories: Respondents who answer "no" to the first question don't get asked this question, and score a 0 for this. If they do answer this question for the Faux Pas Stories, any reasonable answer that makes reference to the faux pas is acceptable. The subject does not have to explicitly mention mental states, as in, "He didn't know about the guy who was sick with cancer, but everyone else did." It is sufficient to say, "Because John is terminally ill," or "because the guy standing right there is married to a lawyer," or "you shouldn't walk into a new apartment and criticize it; you don't know who bought what." This question only gets scored as incorrect if the

person's answer doesn't reflect an understanding of the faux pas, that is, of what would have been offensive.
Examples (from amygdala patients): "The neighbor shouldn't have called her little. Kids like to feel grown up." (Misses the point that Sally is a girl, not a boy.)
"Claire shouldn't tell him she needs her coffee." (Misses the insult to Roger.)
"You shouldn't come into a meeting late." (Doesn't mention the inappropriate joke.)
For the Control Stories: Scoring the Control Stories seems counterintuitive, because you give points for not answering, and take points off for answering. If they correctly say there was nothing inappropriate said, then they should not be answering this question. So, give them one point for each "why" question they do not answer on the Control Stories. For any Control Stories on which they do give an answer, give them zero points, because the answer will be incorrect. Exception: if you are really convinced that their answer is reasonable, give a point.

Intentions
Fourth question: "Why did they say it?" or "Why do you think they said it?"
For the Faux Pas Stories: Respondents who answer "no" to the first question don't get asked this question, and score a 0 for this one. If they do answer this question, any reasonable answer that makes reference to the faux pas is acceptable. As long as the subject's answer indicates that they understand that one of the story characters didn't know something or didn't realize something, it is correct, even if they do not explicitly mention mental states.
Note: This question gets scored as incorrect if the subject seems to think that the person said the awkward thing deliberately. Some more examples, from brain injury patients: "Tim shouldn't order around other customers. He just basically went up to an equal and said, 'On your knees, boy.'" (Doesn't reflect an understanding that Tim mistook Jack for someone who worked at the restaurant.)
"He was trying to put Christine down, make himself one up by gloating." (Doesn't reflect that he didn't know Christine was in the contest.)
"She was trying to make Helen feel jealous." (Looks like a confabulation, and doesn't mention surprise party.)
Some patients also just say, "I don't know," which also gets a zero.
For the Control Stories: Scoring the Control Stories seems counterintuitive, because you give points for not answering, and take points off for answering. If they correctly say there was nothing inappropriate said, then they should not be answering this question. So, give them one point for each intention question (4th question) they do not answer on the Control Stories. For any Control Stories on which they do give an answer, give them zero points, because the answer will be incorrect.

Belief
Fifth question: Did X know that Y?
For the Faux Pas Stories: This question gets at whether they realize the faux pas was unintentional. Respondents who answer "no" to the first question don't get asked this question, and score a 0 for this one. If they do answer it, scoring is straightforward – 1 point if they indicate that the person did not know or did not realize that their statement would be awkward, 0 points if their answer indicates they did not track the story character's knowledge and beliefs.
For the Control Stories: If they correctly say there was nothing inappropriate said, then they should not be answering this question. So, give them one point for each belief question they do not answer on the Control Stories. BUT: this question is to test that they can track knowledge and belief states in the story characters when no Faux Pas is present. So, even though they should not be answering

this question because they should have said, no, no one said anything awkward, it is still informative to score this Belief question. Scoring is straightforward: 1 point if they accurately track the story characters' knowledge and belief states, 0 points if they do not.

Discrepancies between answers to the first question and to the fifth question should be noted.

Empathy
Sixth question: How did X feel? A test of the respondents' empathy for the story characters.
For the Faux Pas Stories: Their answers should reflect feelings of hurt, anger, embarrassment, disappointment, as appropriate. Score 1 point for appropriate answers, and 0 points for answers that are obviously inappropriate. Even in cases of severe TBI where the patient misses the Intention question, I have always found that they answer this one correctly. Still, it will be informative if they get it wrong.
For the Control Stories: If they correctly say there was nothing inappropriate said, then they should not be answering this question. So, give them one point for each Empathy question they do not answer on the Control Stories. BUT: this question is to test that they can track others' feelings in the story characters when no Faux Pas is present. So, even though they should not be answering this question because they should have said, no, no one said anything awkward, it is still informative to score this Empathy question. Scoring is straightforward: 1 point if they appropriately attribute emotional states to story characters, 0 points if they do not.

Story Comprehension – Control Questions
Seventh and eighth questions: All respondents get asked these questions, even if they answer "no" to the first question. These control questions on story comprehension should tell you if the person has gotten confused and forgotten the details of the story, something we have found to be true in patients with more advanced dementia, and some patients with dorsolateral frontal damage. Answers are pretty obvious. These are scored separately from the other questions.
Example correct answers for Faux Pas Stories,
In the story, where was Sally? "At her aunt Carol's house." (I think one subject said, "In the doorway next to her aunt," and I scored it as correct.)
In the story, what had Jeannette given Anne for her wedding? "A crystal bowl," "a bowl."
In the story, what did Roger's wife do for a living? "She was a lawyer."
In the story, what had Jill just bought? "New curtains," "curtains."
In the story, what had Jean West just told people in the meeting? "VP had cancer."
In the story, who won the competition? "Jake."
In the story, where was Jack standing? "By the cashier."
In the story, where was Mike while Joe and Peter were talking? "In the stalls (cubicles)."
In the story, who was Helen's husband throwing a surprise party for? "Helen."
In the story, what kind of pie had Kim made? "Apple."
Dorsolateral frontal patients, for example, often got some of these wrong. One patient said the surprise party was for Sarah's birthday, and that Helen was upset because her husband was throwing a party for another woman, and she wondered if they were having an affair.

Calculating total summary scores:
Break down the scores this way, doing scores on control questions first for both Faux Pas Stories and Control Stories:

1) The 1st 2 scores to report are Correct Control Questions Scores, for the Faux Pas Stories and the Control Stories. To calculate these:
Step 1. For Faux Pas Stories, sum up the number of correct answers to questions 7 and 8, the questions that test for story comprehension. We'll call this score "Correct Control Qs-FP Stories." Perfect score equals 20 points for Faux Pas Stories.
Step 2. For Control Stories, sum up the number of correct answers to questions 7 and 8, the questions that test for story comprehension. We'll call this score " Correct Control Qs-Control Stories."
Step 3. If you want to convert these to % correct, obviously, divide each one by 20.
Step 4. You need to know these scores so that you know how to correct for stories they did not comprehend. Take each story on which they made any errors on control questions, and cross out all their responses to that story. **DO NOT COUNT RESPONSES TO ANY QUESTIONS ABOUT THAT STORY IN ANY SCORES BELOW.**

2) The 2nd score to report is a Faux Pas Detection Score (a ratio). To calculate this:
LEAVE OUT ANY STORIES ON WHICH A CONTROL QUESTION WAS INCORRECT.
Step 1. For each Faux Pas Story, sum up the score on first question (did anyone say something bad?) + score on 2nd question (who said something bad?). Perfect score = 20.
Step 2. For the Control Stories, sum up the score on the first question (did anyone say something bad? They should have responded no). Perfect score = 10.
Step 3. For the Control Stories, sum up the score on the second question (who said something bad?). Scoring this seems counterintuitive, because you give points for not answering, and take points off for answering. If they correctly say there was nothing inappropriate said, then they should not be answering this question. So, give them one point for each “who” question they do not answer on the Control Stories. Perfect score = 10.
Step 4. Sum the scores from Steps 3 and 4. Perfect score = 20.
Step 5. Sum up the score from Step 2 and the score from Step 4.
Step 6. Divide the sum from Step 5 by (Correct Control Qs for FP Stories + Correct Control Qs for Control Stories). Perfect Faux Pas Detection Score = 1.0.

3) The third score to report is the "Understanding Inappropriateness" score, a ratio (why shouldn’t say that) . To calculate this:
LEAVE OUT ANY STORIES ON WHICH A CONTROL QUESTION WAS INCORRECT.
Step 1. For each Faux Pas Story, sum up the score on the 3rd questions (why shouldn’t they have said that?). Perfect score = 10.
Step 2. Scoring the Control Stories seems counterintuitive, because you give points for not answering, and take points off for answering. If they correctly say there was nothing inappropriate said, then they should not be answering this question. So, give them one point for each “why” question they do not answer on the Control Stories. For any Control Stories on which they do give an answer, give them zero points, because the answer will be incorrect. Perfect score = 10 for Control Stories.
Step 3. Sum the scores from Step 1 and Step 2.
Step 4. Take this sum and divide it by the quantity (Correct Control Qs for FP Stories + Correct Control Qs for Control Stories). A perfect score for this “Why?” ratio = 1.0.

4) The fourth score to report is the "Intentions" score, a ratio, the score on answers to "why do you think they said that?". This is called Intentions because it is the only question that gets at the speaker's motivations and intentions. This score appears to be most sensitive to traumatic brain injury. To calculate this score:
LEAVE OUT ANY STORIES ON WHICH A CONTROL QUESTION WAS INCORRECT.
Step 1. Sum up the score on the 4th questions for each Faux Pas Story. Perfect score equals 10 points for Faux Pas Stories.
Step 2. Scoring the Control Stories seems counterintuitive, because you give points for not answering, and take points off for answering. If they correctly say there was nothing inappropriate said, then they should not be answering this question. So, give them one point for each intention question (4th question) they do not answer on the Control Stories. For any Control Stories on which they do give an answer, give them zero points, because the answer will be incorrect. Perfect score equals 10 points for Control Stories.
Step 3. Sum the scores from Step 1 and Step 2.
Step 4. Take this sum and divide it by the quantity (Correct Control Qs for FP Stories + Correct Control Qs for Control Stories). A perfect score for this "Intentions" ratio = 1.0.

5) The fifth score to report is the "Belief" score (a ratio), score on answers to "did X know that Y" questions. To calculate this:
Step 1. Sum up the score on the 5th questions ("did X know that Y") for each Faux Pas Story and each Control Story.
Step 2. Take this sum and divide it by the quantity (Correct Control Qs for FP Stories + Correct Control Qs for Control Stories). A perfect score for this "Belief" ratio = 1.0.

5) The sixth score to report is the "Empathy" score (a ratio), score on answers to "how would X feel" questions. To calculate this:
Step 1. Sum up the score on the 5th questions ("how would X feel") for each Faux Pas Story and each Control Story.
Step 2. Take this sum and divide it by the quantity (Correct Control Qs for FP Stories + Correct Control Qs for Control Stories). A perfect score for this "Belief" ratio = 1.0.

If you want to, you can derive an overall total score for the Faux Pas Recognition Test by summing up the ratios and dividing by what would be a perfect score for each ratio. This total score is not likely to be the most informative measure.

On the following pages are the stories by themselves to print out and put in front of respondents.

SAVE TREES - PLEASE PRINT THIS DOCUMENT DOUBLE-SIDED.
