# Supplementary material for: Large language models for autism: evaluating theory of mind tasks in a gamified environment
Source: Sci Rep. 2025 Oct 6;15:34763. doi: 10.1038/s41598-025-18608-4 (PMC12501279; doi:10.1038/s41598-025-18608-4)
Supplement: Supplementary file 2 — Supplementary Material 2 [file 41598_2025_18608_MOESM2_ESM.pdf]

1 General information for scoring the tasks

The study contains tasks from four different Theory of Mind categories: Faux Pas, Irony, Hinting and Strange Stories. Each category includes both a real and a control task, meaning that all participants complete eight tasks in total.
The participants received the stories for each task in the form of a short animated scene. They saw the characters talking to each other, listened to the story simultaneously (with different voices for each character and the narrator) and read the story as it appeared on the screen. Participants could restart these scenes as often as they liked. Once they felt ready, they could begin answering the questions. They received the questions one after the other and were unable to go back to previous questions or rewatch the scenes.

The type (Yes/No or open text) and number of questions for each task depends on its category. However, two comprehension questions are always asked at the end of each task. These questions establish whether participants have understood and remembered the details of the story. Therefore, no interpretation of the story is necessary; only factual information is required.
For every task, at least one question is asked from each of the following categories: understanding of non-literal meaning/inference of hidden meaning, understanding of social meaning, and prediction of the mental state of the characters involved.

Each answer can be rated as either correct or incorrect. Use your expertise in this field and your intuitive judgment to decide. One point is awarded for each correct answer and zero for each incorrect one. Half points are not possible.

The first sheet of the Excel workbook contains six questions for you to answer. Questions one to four can be answered at the beginning. Questions five and six should be answered once you have finished scoring all the answers. The remaining sheets each contain one participant's answers to all eight tasks. Please enter the points directly into the corresponding column next to the answer.

The following sections provide information on all tasks. This includes the stories, as well as all the questions and their types. It also includes information on whether it is a real or control task, and the maximum points that can be achieved. Additional clarification for scoring the Faux Pas task is also provided.

2 Social Faux Pas Task

2.1 Scoring

Max. points for this task: 9

There are two tasks in this section, each containing nine questions. If participants answer 'No' to the first question, the next four questions (questions two to five) are skipped, but still need to be scored.
For the story containing a faux pas, questions two to five are awarded zero points if they are not answered. If answers are provided, they are scored as normal by checking whether they are correct or incorrect. For the control story (without a faux pas), questions two to five are awarded one point if they remain unanswered and zero points if an answer is provided.

2.2 Task 1:

Faux Pas Task: with Faux Pas

2.2.1 Story:

Sarah is at the supermarket to buy cat food. She got a little black cat called "Daisy" last week. She meets her work colleague Kevin at the supermarket and starts a conversation with him. Sarah says: "Hi! I'm here to buy food for my new cat." Kevin replies: "I hope it's not a black cat, they're ugly and mean bad luck."

2.2.2 Questions:

1. Did anyone say something they shouldn't have said or something awkward? - *Yes/No question*
2. Who said something they shouldn't have said or something awkward? - *Open text question*
3. What did they say that they should not have said? - *Open text question*
4. Why shouldn't they have said it or why was it awkward? - *Open text question*
5. Why do you think they said it? - *Open text question*
6. Is it more likely that Kevin knew or did not know that Sarah has a black cat? - *Open text question*
7. How do you think Sarah felt? - *Open text question*
8. Where did Sarah start the conversation with Kevin? - *Open text question*
9. What did Sarah want to buy? - *Open text question*

2.3 Task 2:

Faux Pas Task: without Faux Pas **- Control Story**

2.3.1 Story:

Today is Caleb's first day at his new job. He has brought cupcakes for all his colleagues and left them in the meeting room. As Caleb walks past an open office door, he hears two colleagues talking to each other inside. One of them says: "Have you tried the cupcakes in the meeting room yet?". The other replies: "No, not yet. I think the new employee brought them."

2.3.2 Questions:

1. Did anyone say something they shouldn't have said or something awkward? - *Yes/No question*
2. Who said something they shouldn't have said or something awkward? - *Open text question*
3. What did they say that they should not have said? - *Open text question*
4. Why shouldn't they have said it or why was it awkward? - *Open text question*
5. Why do you think they said it? - *Open text question*
6. Is it more likely that Caleb's colleagues knew or did not know that Caleb could hear them? - *Open text question*
7. How do you think Caleb felt? - *Open text question*
8. Where were Caleb's colleagues during their conversation? - *Open text question*
9. What did Caleb bring to the office? - *Open text question*

3 Irony Task

3.1 Scoring

Max. points for this task: 5

3.2 Task 1:

Irony Task: with irony

3.2.1 Story:

Hanna is the boss of a large company. As she walks through the office, she sees one of her employees sitting relaxed at his desk watching a movie. Hanna says to him: “I see you’re working particularly hard today.”

3.2.2 Questions:

1. Did Hanna think that her employee was working hard today? - *Yes/No question*
2. What could have been a reason for Hanna to say this? - *Open text question*
3. How could Hanna have felt in this situation? - *Open text question*
4. Who was the boss of the company? - *Open text question*
5. What did the employee do when Hanna walked by? - *Open text question*

3.3 Task 2:

Irony Task: without irony **- Control Story**

3.3.1 Story:

Paul went shopping because he wants to cook dinner with his wife tonight. He bought so much that the whole table is full of food. When his wife sees this, she says: “Great, you bought everything we need.”

3.3.2 Questions:

1. Did Paul’s wife think he had bought everything they needed? - *Yes/No question*
2. What could have been a reason for Paul’s wife to say this? - *Open text question*
3. How could Paul’s wife have felt in this situation? - *Open text question*
4. What did Paul buy? - *Open text question*
5. What did Paul and his wife want to do that evening? - *Open text question*

4 Hinting Task

4.1 Scoring

Max. points for this task: 5

4.2 Task 1:

Hinting Task: with hint

4.2.1 Story:

Clara and her husband recently adopted a puppy called "Max". They are both working on their laptops when the puppy starts whining. Clara says to her husband: "I think Max needs to go outside soon but I have so much work to do."

4.2.2 Questions:

1. What did Clara really mean when she said this? - *Open text question*
2. What reaction could Clara have hoped for? - *Open text question*
3. Did Clara want her husband to do something? - *Yes/No question*
4. What did Clara and her husband do? - *Open text question*
5. What was the puppy's name? - *Open text question*

4.3 Task 2:

Hinting Task: without hint **- Control Story**

4.3.1 Story:

Olivia and Charlie work in the same office. It's already late in the evening and they still have a lot of work to do. Charlie says to Olivia: "I have a really bad headache. I'm going to take a painkiller and I'll be right back."

4.3.2 Questions:

1. What did Charlie really mean when he said this? - *Open text question*
2. What reaction could Charlie have hoped for? - *Open text question*
3. Did Charlie want Olivia to do something? - *Yes/No question*
4. What did Olivia and Charlie do? - *Open text question*
5. What kind of pain did Charlie experience? - *Open text question*

5 Strange stories

5.1 Scoring

Max. points for this task: 6

5.2 Task 1:

Strange stories Task: White Lie

5.2.1 Story:

Thomas has been working in his current job for a very long time. He knows that his boss George doesn't like it when people disagree with him. When Thomas comes into the office, his boss says to him: "Look at the new chairs I've bought for the company. Aren't they nice?" Thomas doesn't like the color and thinks they look uncomfortable. Thomas replies: "Yes, they look great. I particularly like the color."

5.2.2 Questions:

1. Did Thomas mean what he said about the chairs? - *Yes/No question*
2. Why did Thomas say that? - *Open text question*
3. How could Thomas have felt in this situation? - *Open text question*
4. How could Thomas's boss have felt when he heard Thomas's response? - *Open text question*
5. Who did Thomas talk to? - *Open text question*
6. What did Thomas's boss buy for the office? - *Open text question*

5.3 Task 2:

Strange stories Task: no White Lie **- Control Story**

5.3.1 Story:

Melissa is invited to lunch at her friend Camila's house. When Melissa arrives, Camila says to her: "Hi! I've cooked lasagna. I hope you like it?". As a child, Melissa couldn't stand lasagna, but now she enjoys it. Melissa replies: "Yes. I love lasagna."

5.3.2 Questions:

1. Did Melissa mean what she said? - *Yes/No question*
2. Why did Melissa say that? - *Open text question*
3. How could Melissa have felt in this situation? - *Open text question*
4. How could Melissa's friend have felt when she heard Melissa's response? - *Open text question*
5. Who did Melissa talk to? - *Open text question*
6. What did Melissa's friend cook? - *Open text question*
