# Supplementary material for: Large language models for autism: evaluating theory of mind tasks in a gamified environment
Source: Sci Rep. 2025 Oct 6;15:34763. doi: 10.1038/s41598-025-18608-4 (PMC12501279; doi:10.1038/s41598-025-18608-4)
Supplement: Supplementary file 4 — Supplementary Material 4 [file 41598_2025_18608_MOESM4_ESM.pdf]

1 Social Faux Pas Task

1.1 Task 1:

Faux Pas Task: with Faux Pas

1.1.1 Story:

Camila is at the supermarket to buy cat food. She got a little black cat called "Daisy" last week. She meets her work colleague Kevin at the supermarket and starts a conversation with him. Camila says: "I'm here to buy food for my new cat." Kevin replies: "I hope it's not a black cat, they're ugly and mean bad luck."

1.1.2 Questions:

1. Did anyone say something they shouldn't have said or something awkward?
2. Who said something they shouldn't have said or something awkward?
3. What did they say that they should not have said?
4. Why shouldn't they have said it or why was it awkward?
5. Why do you think they said it?
6. Did Kevin know that Camila has a black cat?/ Is it more likely that Kevin knew or did not know that Camila has a black cat?
7. How do you think Camila felt?
8. Where did Camila start the conversation with Kevin?
9. What did Camila want to buy?

1.2 Task 2:

Faux Pas Task: without Faux Pas

1.2.1 Story:

Today is Caleb's first day at his new job. He has brought cupcakes for all his colleagues and left them in the meeting room. As Caleb walks past an open office door, he hears two colleagues talking to each other inside. One of them says: "Have you tried the cupcakes in the meeting room yet?". The other replies: "No, not yet. I think the new employee brought them."

1.2.2 Questions:

1. Did anyone say something they shouldn't have said or something awkward?
2. Who said something they shouldn't have said or something awkward?
3. What did they say that they should not have said?
4. Why shouldn't they have said it or why was it awkward?
5. Why do you think they said it?
6. Did Caleb's colleagues know that Caleb could hear them?/ Is it more likely that Caleb's colleagues knew or did not know that Caleb could hear them?
7. How do you think Caleb felt?
8. Where were Caleb's colleagues during their conversation?
9. What did Caleb bring to the office?

2 Irony Task

2.1 Task 1:

Irony Task: with irony

2.1.1 Story:

Melissa is the boss of a large company. As she walks through the office, she sees one of her employees sitting relaxed at his desk watching a movie. Melissa says to him: "I see you're working particularly hard today."

2.1.2 Questions:

1. Did Melissa think that her employee was working hard today?
2. What could have been a reason for Melissa to say this?
3. How could Melissa have felt in this situation?
4. Who was the boss of the company?
5. What did the employee do when Melissa walked by?

2.2 Task 2:

Irony Task: without irony

2.2.1 Story:

Kevin went shopping because he wants to cook dinner with his wife tonight. He bought so much that the whole table is full of food. When his wife sees this, she says: “Wow, you really bought everything we need.”

2.2.2 Questions:

1. Did Kevin’s wife think he had bought everything they needed?
2. What could have been a reason for Kevin’s wife to say this?
3. How could Kevin’s wife have felt in this situation?
4. What did Kevin buy?
5. What did Kevin and his wife want to do that evening?

3 Hinting Task

3.1 Task 1:

Hinting Task: with hint

3.1.1 Story:

Camila and her husband recently adopted a puppy called “Maxi”. They are both working on their laptops when the puppy starts whining. Camila says to her husband: “I think Maxi needs to go outside soon but I have so much work to do.”

3.1.2 Questions:

1. What did Camila really mean when she said this?
2. What reaction could Camila have hoped for?
3. Did Camila want her husband to do something?
4. What did Camila and her husband do?
5. What was the puppy’s name?

3.2 Task 2:

Hinting Task: without hint

3.2.1 Story:

Melissa and Caleb work in the same office. It’s already late in the evening and they still have a lot of work to do. Caleb says to Melissa: “I have a really bad headache. I’m going to take a painkiller and I’ll be right back.”

3.2.2 Questions:

1. What did Caleb really mean when he said this?
2. What reaction could Caleb have hoped for?
3. Did Caleb want Melissa to do something?
4. What did Melissa and Caleb do?
5. What kind of pain did Caleb experience?

4 Strange stories

4.1 Task 1:

Strange stories Task: White Lie

4.1.1 Story:

Kevin has been working in his current job for a very long time. He knows that his boss Caleb doesn't like it when people disagree with him. When Kevin comes into the office, his boss says to him: "Look at the new chairs I've bought for the company. Aren't they nice?" Kevin doesn't like the color and thinks they look uncomfortable. Kevin replies: "Yes, they look great. I particularly like the color."

4.1.2 Questions:

1. Did Kevin mean what he said about the chairs?
2. Why did Kevin say that?
3. How could Kevin have felt in this situation?
4. How could Kevin's boss have felt when he heard Kevin's response?
5. Who did Kevin talk to?
6. What did Kevin's boss buy for the office?

4.2 Task 2:

Strange stories Task: no White Lie

4.2.1 Story:

Melissa is invited to lunch at her friend Camila's house. When Melissa arrives, Camila says to her: "I've cooked lasagna. I hope you like it?". As a child, Melissa couldn't stand lasagna, but now she enjoys it. Melissa replies: "I love lasagna."

4.2.2 Questions:

1. Did Melissa mean what she said?
2. Why did Melissa say that?
3. How could Melissa have felt in this situation?
4. How could Melissa's friend have felt when she heard Melissa's response?
5. Who did Melissa talk to?
6. What did Melissa's friend cook?
